# Ethical Dilemmas and Privacy Issues in Emerging Technologies: A Review

**DOI:** 10.3390/s23031151

**Published:** 2023-01-19

**Authors:** Lubna Luxmi Dhirani, Noorain Mukhtiar, Bhawani Shankar Chowdhry, Thomas Newe

**Affiliations:** 1 Department of Electronic and Computer Engineering, University of Limerick, V94 T9PX Limerick, Ireland; 2 Confirm—SFI Smart Manufacturing Centre, V94 C928 Limerick, Ireland; 3 Department of Electronic Engineering, Mehran University of Engineering & Technology, Jamshoro 76062, Pakistan

**Keywords:** privacy, ethics, cybersecurity, regulations, standards, emerging technologies

## Abstract

Industry 5.0 is projected to be an exemplary improvement in digital transformation allowing for mass customization and production efficiencies using emerging technologies such as universal machines, autonomous and self-driving robots, self-healing networks, cloud data analytics, etc., to supersede the limitations of Industry 4.0. To successfully pave the way for acceptance of these technologies, we must be bound and adhere to ethical and regulatory standards. Presently, with ethical standards still under development, and each region following a different set of standards and policies, the complexity of being compliant increases. Having vague and inconsistent ethical guidelines leaves potential gray areas leading to privacy, ethical, and data breaches that must be resolved. This paper examines the ethical dimensions and dilemmas associated with emerging technologies and provides potential methods to mitigate their legal/regulatory issues.

## 1. Introduction

### Ethical Dimensions and Dilemmas in Emerging Technologies 

In the current technological era, emerging technologies such as Cloud Computing, Autonomous Vehicles, Artificial Intelligence, Big Data and Machine Learning, and Cybersecurity have enormous potential. These technological advancements raise ethical considerations related to data security and privacy that must be resolved before industries can deploy them in the production environment. Ethical considerations/thinking is based on theories following principles associated to autonomy, justice, beneficence, nonmaleficence and fidelity [1]. *“Ethical thinking is not entirely distinct from thinking in other disciplines but it cannot simply be reduced to them. In particular, ethical conclusions cannot be clearly proved in the way that mathematical theorems can. However, this does not mean that all ethical conclusions are equally valid. After all most philosophers of science would hold that scientific conclusions cannot be unambiguously proved, indeed that they all remain as provisional truths. Some conclusions—whether in ethics, science or any other discipline, are more likely to be valid than others. It is a common fault in ethics courses to assert that there are no rights or wrongs in ethics”* [2]. 

This paper examines the ethical issues, and data privacy and security implications that arise as an outcome of unregulated and non-compliance integrations of these state-of-the-art technologies.

Emerging technologies have featured prominently in the research on technology ethics, which is progressively concentrating on early-stage intervention in technological innovation. Techno Ethics (TE) serves as a multidisciplinary research field that incorporates theories and techniques from various domains including communications systems, sociology, innovation, ethical theories, and principles [3]. For example, the world wide web lacks security and privacy by design while being intended to be a free, accessible, and worldwide service for everybody [4]. Cybercrime is an umbrella term for all illicit activities made possible by access to an IT infrastructure including unauthorized access, unlawful data comparison interception [5], system disruption, digital identity fraud, etc. [6]. The goal of cybersecurity (counterpart to cybercrime) is to assist people in mitigating risks in their systems, networks, and data, ensuring security and privacy. To secure cyberspace, formal and informal resources, including equipment, people, infrastructure, services, policies, training, and technologies are used [7]. As more firms post details to demonstrate their public commitment to ethical ideals while promoting security, discussions regarding ethical standards for emerging technologies are becoming more common [8]. The five major ethical dilemmas currently faced by emerging technologies are (i) data privacy, (ii) risks associated with Artificial Intelligence, (iii) developing sustainable environments, (iv) health implications due to technology use, and (v) infodemic and data weaponization issues. All of these can be addressed using strong policies, regulations, and standards adherence. Unfortunately, there has been limited progress made in the ethical domain in comparison to innovative technological developments/advancements. For the majority of the statutory and regulatory standards (i.e., EU Artificial Intelligence Act (2022), EU Digital Services Act (2022), Digital Markets Act (2022), EU Cyber Resilience Act (2022), etc.) [9,10,11] that have been developed recently for developing a secure, standardized and resilience environment, their impact is yet to be seen. The problem with emerging technologies is that it takes years to understand the types and impacts of the threat landscape, as well as the risks it is susceptible to. One cannot protect an environment without knowing when and which vulnerabilities may or may not occur. These regulations set a roadmap; however, how effective they could be and how aligned they are with the ethical thinking models and theories can only be understood after their implementation.

**Figure 1:**
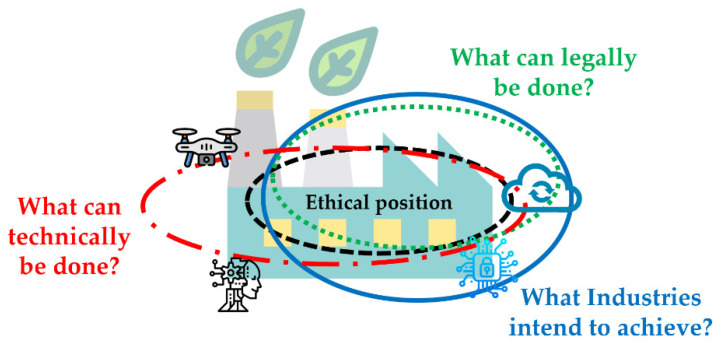
Ethical dilemmas in enabling technologies used in Industrial IoT (diagram adapted from IBM model for ethical analysis [12] and redesigned in context of this paper).

Figure 1 illustrates the struggle between balancing the legal, ethical, technical, and expectations compass [12]. Digitally-transformed industries may tend to be ambitious in terms of outcomes; however, having an alignment between what is legal and what is ethical has been hard to achieve for industries in the past. From that perspective, there is a pressing need to understand the ethical theories (i.e., social contract, utilitarianism, social versus legal liability approach, etc.) [13], and map them with the legal and regulatory compass. Only then there will be a possibility of mitigating the ethical and privacy issues in enabling technologies. 

Only the industries with core competencies are enabled to properly regulate ethical and legal decision-making processes within their environment [12], and this opens up the existing and future manufacturing environment to various regulatory and ethical issues. This paper focuses on ethical and privacy issues related to enabling technologies (i.e., Cybersecurity, Cloud, Autonomous Vehicles, Artificial Intelligence, Big Data, and Machine Learning) and provides insights into the existing regulatory frameworks, policies, and ethical limitations of these technologies. To lawfully and ethically secure these technologies from an end-to-end perspective, the authors examine the privacy and data security metrics (confidentiality, integrity, and availability) from a regulatory and compliance point of view, as there have been various successful cyberattacks due to the poor implementation of regulations and controls in the Industrial IoT environment [14]. Various use-cases (hypothetical and real examples) are discussed throughout this paper to demonstrate the impact of ethical issues in emerging tech and what should be done to mitigate them. 

The paper is structured as follows, Section 2 elaborates different aspects that relate to cyber ethics; Section 3 discusses cloud ethics; Section 4 highlights ethical concerns in autonomous vehicles; Section 5 and Section 6 discuss ethical dilemmas in AI, Big Data, and Machine Learning; Section 7 is based on policy, privacy/compliance, and legal aspects; Section 8 concludes the paper.

## 2. Demystifying Cyber Ethics

Emerging technologies have transformed industries to be more effective and collaborative, and increased dependencies on such platforms. The downside is that, if these technologies are exploited/hacked, they can cause extensive harm to both organizations and people whose data has been compromised, and this is where the ethical concerns (i.e., social contract) fits in. A social contract breach means that an organization’s product or service directly affects the public interest (privacy, safety, and security) [13], for example, a healthcare facility uses an Enterprise Resource Planning (ERP) to manage different aspects of the facility and the IT admin of the healthcare finds a new vulnerability in the ERP that, to fix, requires immediate patching of the system. However, that may take up to 12 h and the ERP will not be operating until the patching is complete. The IT admin knows this will affect the healthcare facility’s in-patient facility and treatments, and, therefore, decides to update the software patch at night when there are less patients and no surgical procedures going on. The admin took a utilitarian approach [15], in which he is choosing a solution that causes the least damage in his point of-view. However, ignoring a patch for later can lead potential hackers straight into the network, causing significant harm (tampering patient data, stealing personal healthcare information, Denial of Service, etc.). The admin’s decision may be based on an utilitarian ethical theory; however, it violates the General Data Protection Regulation (GDPR) Article 32 [16], which states that systems must be patched as soon as the updates are provided, or vulnerabilities are identified. As per GDPR Article 32, patch management is one of the security controls that contributes as an effective baseline security measure, and failure to patch vulnerabilities is considered a regulatory breach that may lead to fines [17]. Article 32 further emphasizes: *“(i) end-to-end encrypted services and pseudonymization for protecting sensitive/personal information (ii) incident response in place (iii) continuously monitoring and evaluating the effectiveness of technical and organizational security measures for identifying new security vulnerabilities/flaws as they emerge/arise (iv) ensuring confidentiality, integrity, availability of data, cyber resilience (having up to date applications and software’s running and patch known network flaws”* [16,18]. With the usage of emerging technologies, it is essential for ethical theories to align with and contribute to the regulatory laws for developing a balance in ethical/moral decision-making processes.

Another dimension that involves an ethical perspective is the different types of hats (i.e., black hat, white hat, etc.). Black hat hackers are cybercriminals having a malicious intent, they look for security vulnerabilities in an environment that can be exploited for stealing data/financial gain. On the other hand, white hat hackers are ethical hackers who perform threat intelligence and pen-testing in a company for identifying and fixing security flaws/weaknesses [19]. The white hat hackers require permission from the company and must oblige the country’s statutory laws which define ethical hacking. The German CDU case acts an eye-opener for zero-day exploits and the need for ethical hacking [19,20]. Gray hat hackers are equally as skilled as black and white hackers, their intent is to look for security vulnerabilities without following the cyber code of ethics [21]. They scan through software vulnerabilities without having the permissions/consent to do so. Gray hat hackers also look for financial benefits in return for full disclosures related to the found vulnerabilities. Red hat hackers [21] are well-known for playing the offense strategy and are motivated by tracking down malicious threat actors for performing counter attacks, and damaging their networks and devices. Red hat hackers are widely known for infiltrating the dark web and launching attacks against malicious/black hat hackers. Blue hat hackers [21] are highly skilled cyber experts hired by enterprises for pen-testing the security posture and improving the cyber defense strategy of their digital environment. Though blue hat hackers are similar to white hats in terms of skillset, they differ based on services offered. Green hat hackers [21] are professionals who wish to pursue careers in cyber hacking; they have limited understanding, experience, and technical knowledge in the domain and are usually found on domains/blogs for asking questions.

With the increasing state-sponsored, Industrial/cyber espionage, counter-back type of cyber-attack scenarios, who decides what is legal and what is not? With a lack of regulations in this space, there are many gray shades. As mentioned above, there are different types of hackers and each may have a different intent. For example, competitors may garner advantages in industrial espionage scenarios, by stealing intellectual property, eavesdropping, etc. Such cyber crimes can be carried out by either a malicious insider or a black/gray hat hacker. To protect industries from these types of cyber crimes, Governments across the world have defined certain permissions and pre-defined limits for industries to follow white hat (ethical) hacking, with the intent to protect their data/organization from potential zero-day attacks and value conflicts in law enforcement brought on by encryption methods [19,22,23]. Though cyber crime and cyber terrorism are both unethical, there is a difference between the two terminologies. Situations where the security of critical infrastructures (electric grid, water supply, healthcare facility ransomware, etc.) [14] is compromised by adversaries, directly affecting human lives, is a breach of the social contract theory and falls under the cyber terrorism category. Presently, there are no defined laws for such situations. A very ethical question arises here: under these circumstances, can the affected/impacted country hold an ethical ground/right to hack-back the state which sponsored the attack? In the last year, the Russian and Ukrainian governments launched various state-sponsored attacks, the ones that affected the people are regarded as cyber terrorism and there is also news of Ukrainian hackers performing hack-back attacks [24]. This ethical question raised above highlights an urgent need for cyber policies and regulations to mitigate escalating cyber warfare/terrorism circumstances. 

The foundation and five pillars of cyber ethics are [1]: justice, nonmaleficence, explicability, beneficence, and autonomy, and the regulations developed to protect the cyber space must conform to these principles. Table 1 sheds light on cyber ethics that relate to privacy, security scenarios, and laws available to protect the environment. 

Researchers in [25,26] discuss various ethical theories, frameworks, and the characteristics that were based on providing ethical solutions for computing, security, etc. Considering emerging technologies, these theories may partially help, as the computer fraud/theft and cybersecurity regulations have evolved in recent times and the focus has moved towards digital forensics. A cyber crime may only be prosecuted if the victim has the ability to provide the digital footprint (evidence of how the crime took place), and this relates to consequential ethics as it is outcome based. The ISC² cybersecurity code of ethics [31] reflects some insights from deontological and virtue ethics as it focuses on the character traits (i.e., honesty, integrity) and the rules/obligations that cyber professionals must follow. These traits and moral characteristics work on individual levels. As an example, one of the key components of virtue ethics is helping others. Based on this, a cyber professional may go beyond means (access controls—i.e., share information or grant access when he/she must not do so), which straight away violates GDPR. Various other contradicting scenarios happen while fully relying on ethical theories. 

With the rising numbers of organized cybercrime attacks, industries are actively hiring and seeking cyber experts and pen-testers to find vulnerabilities/weaknesses in their environment before malicious hackers do. These (white hat and blue hat) hackers [27,28] must abide by the ethical guidelines set by the industry which form a legitimate ground for practicing pen testing, cyber forensics, log analysis, etc. [14,29,30]. This demonstrates the huge impact of cyberattacks on Industry’s information and operational technology environment and provides an understanding for implementing and aligning different cybersecurity and regulatory standards from protecting an environment from cyber data breaches. Digitally-transformed industries enabled with emerging technologies are susceptible to various data security, privacy, and regulatory risks as mentioned in Table 1. To develop a fully resilient Industrial IoT ecosystem, and to mitigate the security (confidentiality, integrity, and availability) and privacy risks, it is essential to understand the flow of data and provide end-to-end security for all three states of data (data-in-use, data-in-transit, and data-in-store). The GDPR principles align and assist in minimizing data security and privacy-based risks by rightfully/legally collecting data, only collecting that which is required, storing it only for a specific time-period (until the purpose is fulfilled), and assuring the confidentiality, integrity, and availability metrics. GDPR abides the ethical obligations, aligns with European statutory regulations, and provides a roadmap of how personal/health data or special category information must be collected, stored, and used.

## 3. Ethical Concerns in Cloud Computing

Cloud Computing has enabled and provided promising outcomes for the industrial IoT environment. The cloud offers different models (i.e., public, private, hybrid, multi-cloud, federated, etc.) and services (Software as a Service, Platform as a Service, and Infrastructure as a Service) [32]. A cloud Service Level Agreement (SLA) is a legal contract agreed between the cloud tenant and the service provider for delivering the promised services. At any point, if the services are not met, the vendor may be subject to a penalty (the contract could be voided) or renegotiated based on new SLA. However, despite having an SLA for controlling the Quality of Service (i.e., reliability, availability, etc.) metrics, the biggest ethical and privacy issues arise when services are processed on third-party premises, without the end-user’s knowledge/consent. Many terms and linked conditions mentioned in the SLA lead to ambiguity and misleading statements [33]. 

By 2025, around 85% of the world’s industrial data will be processed in the cloud [34,35]. The existing models lack the capacity for such resource demands and would rely on federated and brokerage cloud models. However, the federated models lack in terms of standardization, security, governance, risk and control (GRC), trust, access management, incident response, and business continuity. The NIST Cloud Federation Reference Architecture (NIST SP 500-332) [36] only provides a basic understanding of the roles different cloud actors (vendors, carriers, brokers, users, auditors, etc.) perform, a description of technical and service levels, and guidance to ease the barriers for adoption [37].

**Figure 2:**
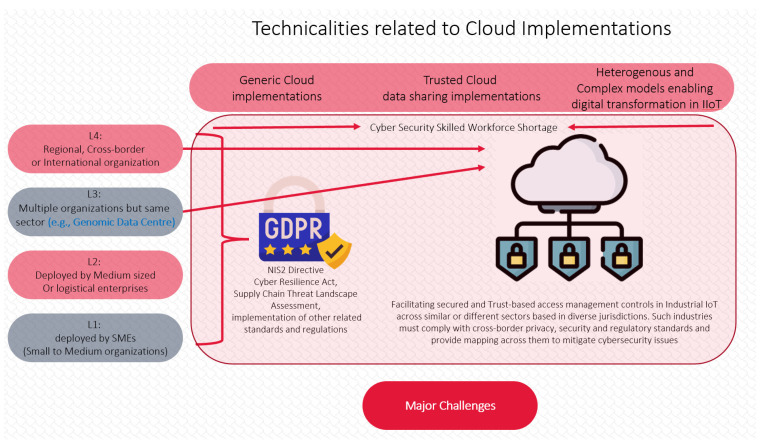
Some of the Cloud Federation challenges addressed by EU H-Cloud [39] (diagram adapted from [39] and redesigned in context of this research).

In 2021, the IEEE P2302 *“Standards for Cloud Federation”* [38] is working on aligning with NIST 800-332; however, that is an on-going project and presently there is no cloud federation model that would provide interoperability and uniform governance. The European-funded Horizon Cloud project [39] that ended in 2022 also demonstrated major data security and regulatory challenges, as shown in Figure 2 below:

Considering these limitations, and taking into account the fact that cloud setups cannot provide end-to-end security or a guaranteed service, is it ethical to process sensitive data on such setups just to save computational costs? Further, if an industry does, the cloud must follow a baseline/minimal security standards and controls to protect the data.

Consider the example of a Genomic datacenter that computes, analyses, and processes DNA structures/patterns and may require huge computing power. At times, the data centers would require sharing the DNA/sensitive information for treatments with other research centers based in different jurisdictions through cloud models. Any breach, or negligence of cloud security standards or policy, would impact all those people receiving treatments, resulting in a breach of the social contract and GDPR as well. In [40], the authors mention ethical challenges related to the privacy and security of genomic data and raises concerns whether the existing compliance and security mechanisms would suffice in securing the data in the transforming nature of emerging technologies. The ethical implications of Cloud Computing are influenced by several technological factors such as: security, privacy, compliance, performance metrics, etc. Table 2, provided below highlights the ethical and privacy considerations in a cloud environment.

Recent cloud data breaches that occurred in 2021 and 2022 [42] raised awareness over different cloud security and vulnerabilities (i.e., (i) Accenture’s LockBit ransomware attack happened because of misconfigured cloud servers that led to data breach, compromising 40,000 customer accounts, causing financial and reputational damage (ii) where as Kesaya’s lack of security implementations for access control, zero trust, remote policies, and multi-factor authentication controls left the cloud SaaS vulnerable and open to zero-day exploits. The number of managed service providers were affected as an outcome of this ransomware attack, leading to three weeks of operational disruption, and financial and reputational damage. The companies impacted by the breaches are well-known, and the cloud service providers were well-known as well, claiming to have strong security mechanisms; yet, a supply-chain type of cyber attack took place). This supports the legitimacy of claims made in [30,31,32,34,41] regarding privacy and regulatory issues, claiming that they are valid and still persist. 

With the increasing adoption of Cloud Computing in different sectors, especially the healthcare industry, it is essential that the appropriate regulatory (GDPR) and security standards controls and kept in place. As cloud is mistaken to be a separate/exclusive entity, the security methods (zero trust, availability, and compliance) used within an industry’s private and public cloud may differ, making it easier for cyber criminals to breach the environment. At present, none of the standardization organizations have provided or released an interoperable cloud standards platform; therefore, the only way to mitigate cloud-based risk would be by developing insights, visibility, and control. Ref. [41] provides a roadmap for understanding the differences at the SLA levels and bridging the gaps between the industrial operational environment and the cloud. However, the gap analysis must be extended as new and innovative technologies are additionally deployed for mitigating the ethical, social, and privacy implications.

## 4. Ethical Dilemmas in Autonomous Vehicles

**Figure 3:**
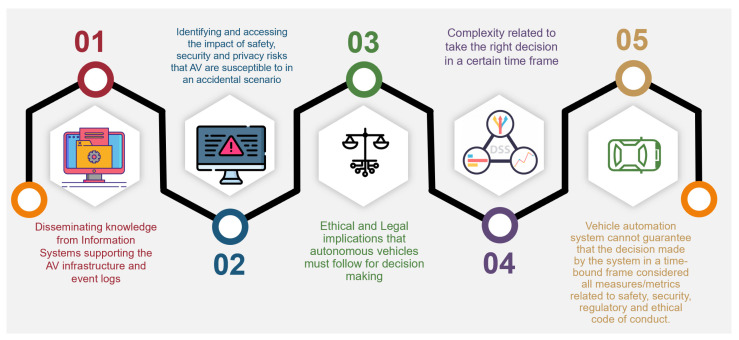
Ethical dilemmas related to AV’s decision making (the diagram is adapted from management strategies for Autonomous Vehicles [19] and redesigned in context of this research).

Fully automated vehicles are already in the development stage and will soon be offered on the market. In recent years, questions related to ethical concerns in autonomous technologies have been increasing. Lawmakers are accustomed to driver assistance, automatic braking, blind spot monitoring, and adaptive cruise control since they regulate traffic safety. These arguments have primarily focused on extreme traffic circumstances portrayed as moral dilemmas, and they are well-documented in the scientific literature, i.e., circumstances where the autonomous vehicle (AV) seems to be required to make challenging ethical choices (e.g., potential hazard situations). Standardization and legalization are needed to help prevent serious issues between society and technology. Also, policies are needed that can verify and validate the ethical behavior of autonomous systems. Once these principles are put in place, they will help to make the system more transparent, effective, and easy to operate. The terms to assist and explain the ethical aspects of automation in vehicles are shown in Figure 3. As autonomous vehicles highly rely on Artificial Intelligence algorithms, they are susceptible to various ethical dilemmas, as shown in Table 3.

The recent Autonomous Vehicles cyber-attacks (i.e., Yandex taxi hack [48], Tesla Model Y [49]) raise similar ethical, privacy, security, and regulatory concerns to those mentioned in [43,44,45,46,47]. At present, one of the biggest concerns is related to the AI-based decision making software used in self-driving vehicles. For example, if the Autonomous Vehicle predicts a collision endangering pedestrians, the AI-based self-learning software (using Big Data and Machine Learning algorithms for predictive analysis in cloud), quickly reroutes and tries to find an alternate path with lesser casualties [50,51]. If this choice is given to the AI software, it may take the path with the least possible casualties (that is single pedestrian), saving the rest of the crowd. Such an approach is called utilitarian ethics. Utilitarian decision making is widely known to be used in warfare situations, where the path for the least fatalities/casualties is optimised. Morally and ethically, it would be impossible for humans to make such a choice: a loss of life is a loss, there is no comparison between a single fatality or multiple. From this perspective, there must be a standardized, compliant, and legal system developed before such autonomous vehicles and devices are implemented in real-time scenarios. 

A graph in [43] presents the automotive industry’s awareness about the ethical concerns regarding self-driving vehicles. Reports from 66 companies based in California were evaluated in the research conducted by [43]. It is interesting to note that the majority of companies focused on: (i) safety and cybersecurity; (ii) sustainability; (iii) human oversight, control, and auditing; (iv) public awareness; (v) privacy; (vi) accountability; (vii) transparency; (viii) ethical design; (viii) legislative frameworks; (ix) dual use problem and military certification. However, none of them addressed the ethical issues related to fairness, non-discrimination, justice, and hidden costs. This form of negligence is in breach of data protection, privacy, and legislative regulations. Also, none of the companies [43] invested in responsible research funding for an emerging technology, which is susceptible to high-risk impact scenarios. Consider if such an AV became involved in an incident or accident, and went unprosecuted due to lack of fairness of data [16], judiciary regulations, and laws in this domain. This may potentially lead to major unrests and promote crimes. Ref. [52] presents an intriguing question related to robot ethics, that is, whether social robots should have certain rights or not. Although the research provides sets of modalities related to robot rights, it mentions that, at this point in time, robots do not possess the necessary capabilities or properties to be considered full moral and legal beings [53,54]. Referring back to cyber attacks mentioned in [48,49], where the hackers took over the command and control, and exploited software vulnerabilities, of Autonomous Vehicles, leading to hours of traffic jam, presents the level of escalated cyber risks autonomous devices are susceptible to and the impact they may have. The authors agree with the recommendations of [53,54] in terms of autonomous decision making: such devices must only be enabled once the potential risks have been realised, controlled, and mitigated. They should also be bound around standardised regulatory and ethical guidelines/bindings.

## 5. Understanding Ethical Artificial Intelligence (AI)

Artificial Intelligence (AI)-based technologies have accomplished incredible things such as machine vision, medical diagnosis, and Autonomous Vehicles. They hold immense potential for improving societal progress, economic expansion, and human welfare and security [55]. Despite this, industries, societies, and communities face serious hazards because of the low degree of interpretability, data inaccuracies, data protection, privacy laws, and ethical issues with AI-based technologies. One of the biggest challenges in this domain is developing AI that is compliant with moral and ethical requirements. To deal with this, industries must look at both dimensions (AI Ethics and ways to develop Ethical AI). AI Ethics refers to the study of the moral principles, regulations, standards, and laws that apply to AI, following the fundamental principles related to: transparency, respect for human values, fairness, safety, accountability, and privacy [55,56]. These principles are similar to the ones the European GDPR [56] provides. The EU AI Act, passed in 2022, aims to develop a legal framework for AI to promote trust and mitigate potential harm that the technology may cause. However, the Members of the European Parliament have addressed their concerns associated with fundamental rights assessment for high-risk users this year. As per the AI Act, a detailed plan for risk impact assessment related to various threat scenarios, potential breaches (i.e., compliance, AI-cybersecurity, etc.) must be provided [10]. As the AI Act is still a work in progress, it is essential to understand the principles on which AI ethics is based and how Ethical AI could be developed.

### 5.1. Transparency

AI-based algorithms and techniques must be transparently designed, with a thorough description as well as a valid justification for being developed, as they play a crucial role in tracking the results and ensuring their accordance with human morals so that one can unambiguously comprehend, perceive, and recognize the designs decision-making mechanism. Twitter serves as an eye-opener here, in 2021 the company faced huge criticism for using AI algorithms to assess racial and gender bias [57]. Twitter is now making amends to mitigate the damages caused by the algorithm and implement the six fundamental attributes of AI Ethics. Considering an industrial/Cyber-Physical System (CPS) environment, transparency is essential for both humans and universal machines.

### 5.2. Respect for Human Values

AI inventions are obliged to uphold human values and positively affect the progress of individuals and industries, as well to assure to protect sensitivity toward cultural diversities and beliefs.

### 5.3. Fairness

Fostering an inclusive environment free from discrimination against employees based on their gender, colour, caste, or religion is essential (including team members from various cultural backgrounds helps to reduce prejudice and advance inclusivity). In the past, AI algorithms have been criticized for profiling healthcare data, employees’ resumes, etc. Considering this from a GDPR perspective, fair use of data in the European jurisdiction is mandatory. Since the fairness aspect maps across AI fairness and GDPR fair use of data, they must be aligned.

### 5.4. Safety

Safety relates to both the security of user information and the welfare of individuals. It is essential to recognize hazards and focus on solutions to eliminate such issues. The users’ ownership over the data must be protected and preserved by using security techniques such as encryption and giving users control over what data are used and in what context. This also aligns with the scope of GDPR.

### 5.5. Accountability

Decision-making procedures should be auditable, particularly when AI is handling private or sensitive information such as copyright law, or identifying biometrics information or personal health records.

### 5.6. Privacy

Protecting user privacy while using AI techniques must be kept as the highest priority. The user’s permission must be obtained to utilize and preserve their information. The strictest security measures must be followed to prevent the disclosure of sensitive data.

Lessons must be learnt from Google’s project Nightingale and Ascension [58] lawsuits which were an outcome of gathering personal data and raised privacy concerns in terms of data sharing and the use of AI. There are various dilemmas when it comes to the applicability of AI. As an example, AI’s implementation in self-driving vehicles has raised huge ethical concerns because, when its designed software was based on a utilitarian approach, in a crash type of situation it would opt for the option with the least casualties; however, when it was programmed based on the social contract theory, the autonomous vehicle could not make a decision as it kept looking for pre-set conditions in loops which ultimately resulted in an accident, as it did not move itself away from the hazard situation [50]. This is one of the biggest challenges, to enable AI to think similarly to humans and have the same ethical and moral conduct; however, with the growing autonomous and self-driving industry there is no going back. Therefore, the only means to control ethical issues related to AI would be to fully develop the standards and regulations. As the authors mentioned earlier, risk impact assessment is merely a means for damage control (analyzing the impact of a breach or vulnerability if exploited). As well, for the cybersecurity threat landscape [14], where the threat actors are constantly evolving, regulating AI—where number of implications are yet to be realized, only best practices and following existing standards and policies can mitigate risks associated to AI deployments in the Industrial environment.

Table 4 elaborates ethical guidelines and existing directives for AI. The authors suggest that a gap analysis of the similarities between them could assist in bridging the compliance/regulatory gaps in the Industrial environment.

A technology that is agile, intelligent and value-driven has already set its course towards digitally transforming the environment. International policy-makers [9,10,11], professional bodies [59,60,61], and industries [61,62,63] have realised the need for regulation, encouraging smooth and higher deployments of AI in the Industrial environment.

The three frameworks [16,57,58] developed by different professional standards/regulatory bodies have few attributes in common; however, a complete mapping or interoperability between the ethical frameworks was not provided. This becomes a potential issue when industries tend to implement a standardized approach. Another issue arises when industries have different manufacturing regions/setups across the world (Europe, USA, and China) and are subject to different jurisdictions, data regulations, and compliance. In circumstances where a production environment deploys different AI regulatory frameworks, it will make the dissemination of information across the digital factory, supply chain, and data classification a complex process. Industry 5.0 is value driven and its vision may only be achieved by mapping synergies across the ethical, technical, innovative, and sustainable domains. The guidelines provided by the EU in [9,10,11] have been the first ones to take initiative in shaping European Digital Strategy, developing standardized regulatory and legal frameworks for AI and mitigating the potential risks. Aligning the AI deployments with the provided Act and security controls [14] is the only regulated way for now, imbibed with the AI ethical principles (i.e., privacy, accountability, fairness of data, transparency), that contribute and map with GDPR principles as well. However, as discussed earlier, it is important to note that AI depends on various technologies (i.e., Big Data, Machine Learning, etc.). If any of these technologies have security gaps, it may lead to potential breaches in the AI domain as well; therefore, the adapting industries must make sure that their ethical and legal framework is compliant and reflects across the interconnected emerging technologies.

## 6. Ethical Concerns Related to Big Data and Machine Learning

**Figure 4:**
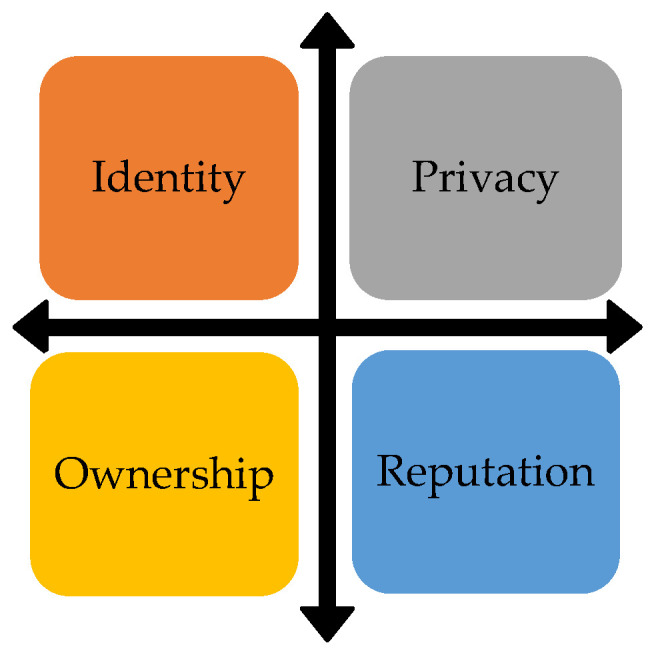
Ethical aspects of Big Data, ML, and AI; a few of these may relate to other emerging technologies as well.

Big Data is a computational paradigm that allows for gathering and utilizing enormous volumes of data characterized by volume, diversity, velocity, authenticity, variability, and complexity, enabling the industrial environment to quickly access, evaluate, and use information. It can also allow them to obtain data that violates an individual’s rights. It can occur either intentionally or unintentionally [64]. This leads to a variety of Machine Learning (ML)- and Big Data-specific ethical [65,66,67,68,69] and privacy challenges (i.e., immoral behavior and producing dark patterns if ethical principles are not carefully implemented). AI, ML, and Big Data set the paths for innovation and digital transformation; however, if these emerging technologies do not manage the data risks appropriately, they will be susceptible to various risks such as: identity, data privacy, and reputational damage. GDPR has facilitated controlling the number of data risks related to data ownership, data minimization, accuracy, purpose limitations, compliance, etc. ML and Big Data share similarities with Ethical AI in terms of the first three attributes (identity, privacy, and reputation) shown in Figure 4 below:

Table 5 provides and further explores the ethical aspects related to identity, privacy, ownership, and reputation.

Regarding Big Data, the 5V’s (volume, velocity, value, veracity, and variety of data) are essential to produce valuable information from the given data [64]. Deploying these attributes in an Industrial IoT (IIoT) environment also brings the responsibility for industries to assure customers regarding their safe and ethical creation, collection, storage, and transmission of data. As the volume of data grows, complexity related to the data integrity increases as well. Ref. [68] states Big Data veracity (accuracy) errors incur due to the following issues: unauthentic data collection, missing information, and representativeness. As industries collect data from different sources, each source may have or follow a different format for data collection leading to incomplete, vague, and inconsistent data. Ref. [69] cites *“the task of keeping analytics ethical-compliant becomes increasingly challenging because the legal framework surrounding data analytics operations is often vague and poorly defined. Moreover, the vague legal frameworks are not necessarily in-line with end-users ethical values. Besides, from an industrial perspective, data analytics operations have pressure from the industry to meet business goals and engineers to stay within the technological possibilities. Therefore, industries have to spend a considerable amount of time to design their operations ethically”*.

Having ethical decision-making builds trust and reputation as it abides with the ethical social contract theory and provides a competitive advantage to industries. Dealing with a massive amount of data requires data security and regulatory checkpoints at all ends (i.e., while data is stored, while data is in transit, and while data is in use). The authors reviewed recently published papers [62,63,64,65,66,67,68] and identified that only GDPR was being implemented by industries from a compliance/regulatory perspective, and few security controls have been implemented; however, as each industry is diverse and may have a different production environment, they may be susceptible to different data privacy/security risks from an ethical perspective. Such considerations were partially taken into account.

## 7. Policy, Privacy/Compliance, and Legal Aspects

This section discusses the emerging technologies from the policy, privacy, and legal perspectives. Emerging Technologies refer to the most notable, cutting-edge, and innovative technologies for digital transformation, for which various threat and potential risk scenarios must be mitigated to establish ethical standards for enabling technologies. The Precautionary Principle (PP) formalizes the use of caution while developing new technologies, especially when there is a chance that doing so could have negative consequences on the environment and health impacts [70]. *“No other safety principle has been so hotly debated”* as the PP, according to some who claim it *“stifles innovation by placing unrealistic criteria on the safety of new technologies”* [71]. To ensure that human values are supported and honored by the design, one of the more popular approaches is *“value-sensitive design,”* which aims to discover pertinent human values during the development and research phases of technology [72]. There are numerous additional values at risk when people, technology, and the environment interact, such as respect for privacy, environmental sustainability, accountability, and many others. Technology Assessment (TA), in addition to the PP, is among the most well-known techniques for handling uncertainty. By evaluating and investigating ideas, designs, plans, or visions for future technology, Technological Ethics is a technique that *“creates and assesses prospective knowledge about the future consequences of technology”* [73]. The New and Emerging Science and Technology (NEST) ethics framework accomplishes three factors. It begins by outlining the promises and anticipations associated with a revolutionary system. Next, it outlines important arguments that could be made against or for these predictions, such as those relating to efficiency and efficacy. It also lists numerous traditional ethical objections, such as those relating to rights, damages, responsibilities, equitable distribution, and the positive experience, as well as other parameters. Lastly, it recognizes a series of arguments and refutations pertaining to the advantages and disadvantages of the technology that can be used to predict how the moral discussion of technological advances might proceed. The rules governing the use of information, proof, artistic creations, and inventions apply to the legal difficulties relating to emerging technologies. The four laws include:

- Privacy law governs the gathering, use, processing, and disclosure of personal information. Most privacy regulations define personal information as data that identifies a person or makes it possible for a person to be identified;
- Evidence law controls how evidence is presented in court proceedings;
- Copyright law regulates issues related to ownership and artistic creations;
- Patent law governs ownership of intellectual property (IP).

These laws have been laid out to form a code of legal, regulatory, and moral conduct. The emerging tech issues related to privacy and ethics will only increase and do more harm than good, until and unless an ethical code of conduct aligned with regulations and legislations is made mandatory for industries to comply with. The EU’s digital strategy has actively been involved in developing standards and regulations for new technologies. Some of those regulations are: the EU Cyber Resilience Act, NIS2D (Network and Information Security Directive) [74], GDPR, AI Act, Digital Markets and Services Act, Digital Operational Resilience (DORA) [75], EU Cybersecurity Strategy [76], EU Cybersecurity Act [77], EU Toolbox [78], etc. The main objectives of these regulations are to build trust, transparency, authenticity, accountability, responsibility, and ease, and increase business operations across EU which are enabled with safe and secured data sharing. It is essential for industries to understand the digital capacities and interoperability between enabling technologies and the above-mentioned frameworks. A dynamic risk and incident assessment for each of these technologies must be in place, as they vary in terms of features and functionality. Despite the different roles each technology plays, it must comply in terms of data privacy, security, and ethics to provide a successful industrial environment. Regarding the privacy and security landscape, the regulations have incorporated ethical principles within them, reducing the dilemmas (as shown in Figure 1). However, in today’s time, these regulations can provide a roadmap towards building a secure and ethical environment; however, they cannot guarantee that a data breach may not occur. This is where the end-users will have to forge alignment and implement best practices with their business/industrial environment.

## 8. Conclusions

This paper reviews the ethical issues, challenges, compliances, rules, and regulations for emerging technologies, including Cybersecurity, Cloud, Autonomous Vehicles, Artificial Intelligence, Big Data and Machine Learning through a comprehensive literature review. A synopsis of the ethical dilemmas in different use-case examples is provided; next, the authors look into the technical standards (i.e., privacy, compliance, security, etc.) and provide an understanding of how the issues arising from enabling technologies must be addressed and aligned in terms a of regulatory and ethical code of conduct. With the continuously evolving technologies, it is hard to set a firm policy, or standard or ethical ground, as each ethical dilemma in tech is different and must be subjected to a different social, ethical, and legal solution. An analysis of different aspects of ethical decision making is provided. Ultimately, this paper provides insights for novices on developing an ethical, legal, and standardized industrial environment deploying emerging technologies.

## Figures and Tables

**Table 1:** Cyber ethics: legal, privacy, and security risks.

Title	Overview	Ethical, Legal, Privacy and Security Risks
Predicting and Explaining Cyber Ethics with Ethical Theories [25]	Mentions prominent ethical theories employed to forecast and defend choices in the context of computer ethics, security, academic integrity, and intellectual property right. The research represents conceptual and predictive models to examine a group of theories. The findings indicate that computer ethics, internet security, and intellectual integrity are most significantly predicted by consequential ethics. Academic integrity is not considerably predicted by deontological ethics, but it is found to be significantly predicted by intellectual property rights.	The following ethical theories have been suggested as the most relevant to technological applications, throughout the research. (i) Consequentialism (outcome-based) (ii) Deontological ethics (duty-based) (iii) Virtue Ethical Theory (character-based) These theories do not align with GDPR and hence would create more issues if followed.
A Principlist framework for cybersecurity ethics [26]	Addresses the moral concerns brought forth by four prominent cybersecurity scenarios including system administration, malware, distributed denial of service attacks (DDoS), and packet sniffing. The case study in this paper presents a principlist framework for analyzing cyber ethics, enhancing ethical knowledge and sensitivity of cybersecurity professionals.	The framework is based on these principles: explicability, beneficence, autonomy, non-maleficence and justice. Discusses the foundation of cyber ethics that may assist in decision-making.
Legality of Ethical Hacking [19]	Discusses the legal grounds over which industries have the right to practice ethical hacking. e.g., a stolen Intellectual Property (IP) or trade secret may incur massive financial, reputational and legal cost damages (worth millions of dollars).	Ethical hackers also known as white hat hackers are employed to find security vulnerabilities and weaknesses in an industry’s environment and provide measures to protect the cybersecurity posture.
Cyber Security and Ethical Hacking: The Importance of Protecting User Data [27]	Elaborates ethical hacking strategies for securing privacy using international security standards and techniques. Preventative measures for cyber threats are also discussed.	Confidentiality, integrity and availability of data are the key elements for the information security standard (ISO 27001). It is also essential to have a systematic way for analyzing and assessing the cyber strategy. This is where various frameworks such as: NIST Cybersecurity Framework (CSF), Risk Management Framework (RMF), etc. fit in.
Industrial IoT, Cyber Threats, and Standards Landscape [14]	Provides insights on Industry 4.0’s cybersecurity threat landscape and provides a roadmap for aligning data security standards and mitigating cybersecurity issues in information and operational technology. A summary of various security standards at the Informational and Operational Technology levels is provided.	For enabling end-to-end (E2E) data security, it is essential to align cybersecurity, compliance, and privacy standards. Each industry may have a different operational environment and will require a unique cybersecurity strategy for risk management and threat intelligence.
Exposing Security and Privacy Issues on Cyber Physical Systems (CPS) [28]	Suggests privacy and security precautions that Industrial and automotive environment should take for preventing unexpected repercussions for apps and users.	Focuses on the GDPR principles (lawfulness, purpose limitation, data minimization, accuracy, storage limitation, security and accountability) for securing data privacy in CPS.
Addressing the Security, Privacy, and Trust Issues in IoT-Enabled CPS [29]	Focuses on security and privacy concerns brought up by IoT-enabled CPS systems.	Specifies security attributes and factors that impact the IoT-enabled cyber physical environment.
Stuxnet, Schmitt Analysis, and the Cyber *“Use-of-Force”* Debate [30]	Several analytical approaches were considered to determine the cyber actions use of force. The theories used provided insights on how cyber operations may lead to physical harm or damage be considered as a catalyst/reactive force.	The analytical factors demonstrated an impact in terms of severity, invasiveness, presumptive legitimacy, directness, measurability, responsibility, immediacy.

**Table 2:** Ethics and privacy in Cloud Computing.

Title	Overview	Ethical and Privacy Risks
Ethical Considerations in Cloud Computing Systems [34]	Elaborates the relationship between ethics and the Terms and Conditions (T&C) guidelines. It provides a comparison of ethical concerns with cloud-based applications versus regular web-based alternatives.	Privacy, security, compliance, monitoring, QoS metrics issues arising due to lack of pre-defined rules end-user cloud SLAs.
Tenant-Vendor and Third-Party Agreements for the Cloud: Considerations for Security Provision [32]	Discusses data-integrity and security implications in hybrid cloud tenant-vendor-subcontracting scenario, highlights SLA limitations and provides solutions to mitigate these issues.	Highlights data integrity, compliance, GDPR implications and cloud virtualization based risks. These ethical, privacy and vendor lock-in issues are an outcome of ambiguous and inconsistent vendors service level agreements.
Data Privacy and Trust in Cloud Computing [41]	Explores some of the identified obstacles to cloud trust and suggests some potential solutions. Also proposes a high-level framework for examining responsibility (trust repairing) and assurance (trust building) in the cloud and argues for a better integrated multi-stakeholder approach to convince research in this complex environment.	Following data risks were demonstrated: relational, performance-based, regulatory and compliance based, technological risks, raising trust based concerns related to cloud deployments.
Hybrid Cloud SLAs for Industry 4.0: Bridging the Gap [33]	Addresses lack of alignment of Cloud Computing in Industry 4.0 and its impact on the industrial environment. It also provides a roadmap for mitigating the gap issues.	Mentions lack of data integrity, compliance, trust-method and standards issues that arise due to unalignment between the industrial and cloud environment. Each industry varies in terms of functionality and operations, in such scenarios generic cloud or security standards may not protect the environment. The only way to resolve these issues is by performing a gap analysis between the cloud and enabling technologies deployed in the industry. Once the gaps/flaws are identified, security controls can be applied to neutralize/mitigate the risks. This approach will also help in building cross platform convergence between the emerging technologies.

**Table 3:** Ethical dilemmas in Autonomous vehicles.

Title	Overview	Ethical Concerns
The Future of Transportation: Ethical, Legal, Social and Economic Impacts of Self-driving Vehicles in the Year 2025 [43]	Summarises the numerous ethical, legal, societal, and economic effects that may arise while implementing self-driving vehicles by 2025, including concerns about individuality, confidentiality, accountability, privacy, and data security.	Security and damage prevention, autonomy, responsibility, rights, data privacy insurance, and discrimination.
Ethical issues in focus by the autonomous vehicles industry [44]	Reviews AVs ethical stories published in scientific papers and business reports by organizations holding California AV testing permits.	Raises concerns over cybersecurity, safety, accountability, human carelessness, and control concerns.
Self-Driving Vehicles—an Ethical Overview [45]	Offers a thorough discussion on the ethical concerns that realistic self-driving car technologies offer. Highlights strong arguments in favor of and against driverless cars and safety necessities for the road traffic system.	Responsibility, public attitudes, safety, control, information, and social Justice
The Future of Automated Vehicles in Canada [46]	Outlines the Transportation and Road Safety Ministries report on adoption of AV on public roads having short, medium, and long-term policy ramifications. Also identifies possibilities, limitations, and strategies for fostering collaboration both domestically and abroad.	The following issues were mentioned: road safety, standards and rules cannot be created separately, innovation needs to be encouraged, privacy issues, education and awareness, technological expertise, traffic laws and requirement of updated traffic rules.
Cybersecurity Challenges in the uptake of Artificial Intelligence in Autonomous Driving [47]	Discusses the key ideas underlying the cybersecurity of AI for autonomous vehicles.	The following issues were summarized: lack of knowledge and data validation techniques for the AI system, encryption and authentication issues, and flaws in security design.

**Table 4:** Ethical Issues and Directives for AI.

Title	Overview	Ethical Guidelines and Directives
Ethics guidelines for trustworthy AI—Publications Office of the EU [59]	Proposes a hierarchy of ethical standards for reliable AI and provides a framework that includes a systematic approach for resilient AI, ethical AI, and legal AI. It also focuses on respect for individual freedom, avoiding violence, justice, and explicability that serve as the foundation of the paradigm.	Provides policies on human intervention and control, technological reliability and security, management of data and privacy, equal protection, transparency, individual and community safety, and liability.
IEEE [60]	Addresses both arguments in favor of the beneficial consequences as well as cautions regarding potential privacy violation, prejudice, skill loss, economic repercussions, protection of vital infrastructure, and everlasting impacts on society.	Individual rights, security, data accountability, efficiency, compliance, awareness of abuse, and competency.
Artificial Intelligence Policy: A Primer and Roadmap [61]	Provides a conceptual framework based on AI policy, intended to assist decision-makers, investors, academics, and students in comprehending the current policy landscape surrounding AI and the issues it poses.	Fairness and Justice, use of force, security and authentication, sovereignty and concealment, taxes, and labor mobility.
AI-based applications and algorithms used in an Industrial IoT (IIoT) environment [62,63] demonstrated that none of the applications and algorithms had data privacy controls in place leading to ethical and legal issues.		
Smart Helmet 5.0 for Industrial IoTs using AI [62]	Presents a comparative analysis of the latest AI-based supervised learning approaches and proposes the use of a Deep Convolutional Neural Network (ConvNet/CNN) to identify potential professional threats.	Threat identification was performed using an AI algorithm but the Smart Helmet 5.0 did not provide data privacy
Industrial IoT and unsupervised deep learning enabled real-time occupational safety monitoring in cold storage warehouses [63]	Proposes a structure for a smart system using the IIoT and digital twin (DT) systems, to implement real-time workplace safety surveillance in the warehouse and guarantee synchronized cyber-physical areas for data provenance and accessibility.	The implementation involved surveillance and lacked securing data privacy in the workplace.

**Table 5:** Ethical and Privacy issues in Big Data and ML.

Title	Overview	Ethical Issues
**Integrating Ethics within Machine Learning Courses** [65]	Identifies and discusses prospects for ML courses for incorporating ethical considerations.	The following ethical concerns in ML were identified: indecisive, cryptic, and misconceived evidence, inequitable results, transformational impacts, and identifiability.
**Privacy Issues and Data Protection in Big Data: A Case Study Analysis under GDPR** [66]	Investigates various data protection and privacy-preserving strategies in the framework of Big Data analysis, as well as the present state of legislative restrictions.	Focuses on the legal and regulatory issues (i.e., data security, anonymization, Quasi identifier, unequivocal identifier, automated decision making, etc.) that may arise in terms of lawfulness, fairness, and clarity while gathering and handling private data.
**An ethical framework for Big Data and smart cities** [67]	Elaborates ethical considerations related to smart cities and Big Data. The conclusions and evaluation regarding Big Data are validated in terms of the rapid expansion, novelty, strategic capabilities, and authenticity of the ethical framework.	Smart cities involving Big Data must apply strong regulatory, ethical, and security controls as they are susceptible to data protection, privacy, integrity, personal information and reputational damage implications.

## Data Availability

Not applicable.
